# Superficial zone cellularity is deficient in mice lacking lubricin: a stereoscopic analysis

**DOI:** 10.1186/s13075-016-0967-4

**Published:** 2016-03-14

**Authors:** Naga Padmini Karamchedu, Josef N. Tofte, Kimberly A. Waller, Ling X. Zhang, Tarpit K. Patel, Gregory D. Jay

**Affiliations:** 1 Department of Orthopedics, Rhode Island Hospital; Warren Alpert Medical School of Brown University, Providence, RI USA; 2 Department of Orthopedics and Rehabilitation, University of Iowa Hospitals and Clinics, Iowa City, IA USA; 3 Department of Emergency Medicine, Rhode Island Hospital, Providence, RI 02903 USA; 4 Department of Engineering, Brown University, Providence, RI USA

**Keywords:** Articular cartilage, Chondrocyte, Lubricin, Apoptosis, Osteoarthritis, Stereoscopy, Friction

## Abstract

**Background:**

Lubricin, a mucinous glycoprotein secreted by synoviocytes and chondrocytes plays an important role in reducing the coefficient of friction in mammalian joints. Elevated cartilage surface friction is thought to cause chondrocyte loss; however, its quantification and methodological approaches have not been reported. We adapted a stereological method and incorporated vital cell staining to assess cellular loss in superficial and upper intermediate zones in lubricin deficient mouse cartilage.

**Methods:**

The femoral condyle cartilage of the intact knees from lubricin wild type (*Prg4* ^+/+^), heterozygote (*Prg4* ^+/-^), and knockout (*Prg4* ^-/-^) mice was imaged using fluorescein diacetate (FDA), propidium iodide (PI), and Hoechst staining, and confocal microscopy. Three dimensional reconstructions of confocal images to a depth of 14 μm were analyzed using Matlab to determine the volume fraction occupied by chondrocytes in cartilage of both medial and lateral femoral condyles. Living chondrocyte volume fraction was defined as FDA stained chondrocyte volume/total volume of superficial + upper intermediate zone. Living and dead (total) chondrocyte volume fraction was defined as FDA + PI stained chondrocyte volume/total volume of superficial + upper intermediate zone. MicroCT provided an orthogonal measure of cartilage thickness. Immunohistology for activated caspase-3 and TUNEL staining were performed to evaluate the presence of apoptotic chondrocytes in *Prg4* mutant mice.

**Results:**

Living chondrocyte volume fraction of the medial femoral condyle was significantly lower in *Prg4* ^-/-^ mice compared to *Prg4* ^+/+^ (*p* = 0.002) and *Prg4* ^+/-^ (*p* = 0.002) littermates. There was no significant difference in medial condyle chondrocyte volume fraction between *Prg4* ^+/+^ and *Prg4* ^+/-^ mice (*p* = 0.82). No significant differences were observed for the chondrocyte volume fraction for the lateral condyle (*p* > 0.26). Cartilage thickness increased in the medial condyle for *Prg4* ^*-/-*^ mice compared to *Prg4* ^*+/+*^ (*p* = 0.02) and *Prg4* ^*+/-*^ (*p* = 0.03) littermates, and the lateral condyle for *Prg4* ^*-/-*^ mice compared to *Prg4* ^*+/+*^ (*p* < 0.0001) and *Prg4* ^*+/-*^ (*p* < 0.0001) littermates, indicating that a multi-dimensional increase in cartilage volume did not artifactually lower the chondrocyte volume fraction in the medial condyle. Significantly higher number of caspase-3 positive cells were observed in the superficial and upper intermediate zone cartilage of the medial femoral condyle of *Prg4* ^*-/-*^ mice compared to *Prg4* ^*+/+*^ (*p* = 0.01) and *Prg4* ^*+/-*^ (*p* = 0.04) littermates, and the lateral femoral condyle of *Prg4* ^*-/-*^ mice compared to *Prg4* ^*+/+*^ (*p* = 0.02) and *Prg4* ^*+/-*^ (*p* = 0.02) littermates. There were no significant differences in TUNEL staining among different *Prg4* genotypes in both condyles (*p* > 0.05 for all comparisons).

**Conclusions:**

Increased Caspase-3 activation is observed in *Prg4 *deficient mice compared to *Prg4 *sufficient littermates. Absence of *Prg4* induces loss of chondrocytes in the superficial and upper intermediate zone of mouse cartilage that is quantifiable by a novel image processing technique.

**Electronic supplementary material:**

The online version of this article (doi:10.1186/s13075-016-0967-4) contains supplementary material, which is available to authorized users.

## Background

Lubricin, a mucinous glycoprotein secreted by synoviocytes and chondrocytes [1–3], plays an important role in reducing the coefficient of friction in mammalian joints [4, 5]. Lubricin knockout (*Prg4* ^-/-^) mice [6] lack lubricin and have increased joint friction during ex vivo loading compared to wild-type mice [4]. The same is true of patients with the camptodactyly-arthropathy-coxa vara-pericarditis syndrome (CACP). The cartilage of *Prg4* ^-/-^ mice appears normal at birth, but exhibits friction-induced wear by two weeks of age [4]. In addition to protecting against superficial zone damage [4], lubricin reduces shear-induced cartilage strain [6–8] thereby protecting chondrocytes [9] embedded in cartilage from strain induced apoptosis [8].

Understanding cellular loss in the absence of lubricin may be relevant to other more common rheumatic and orthopedic conditions which display synovitis. The inflammatory component of joint diseases results in a downregulation of lubricin, which is orchestrated by cytokines such as IL1 and TNFα [9–12]. This deficiency is particularly important in understanding post-traumatic osteoarthritis following anterior cruciate ligament (ACL) disruption, for example, where synovial fluid lubricin levels remain diminished for up to a year [13]. Inadequate lubricin has also been observed in rat osteoarthritis (OA) models where lubricin is undetectable at the cartilage surface four weeks following ACL transection [11]. The progression of traumatized cartilage to OA occurs over several years, involves early damage to the cartilage surface, and the loss of chondrocytes, before characteristic joint remodeling [14–17]. Loss of chondrocytes has been attributed to apoptosis, caused by elevated friction in the *Prg4* ^*-/-*^ mouse [18, 19] and in inflammatory surgical models of OA [20]. A combination of techniques is necessary to confirm apoptosis [21, 22]. We probed for caspase-3 activation and DNA strand breaks. Linking this cellular loss to a lack of lubricin has motivated the re-examination of mice with mutations in *Prg4*.

Since *Prg4* ^-/-^ mice are congenitally deficient in their lubricating ability, we hypothesized that shear-stress induced chondrocyte apoptosis [8] would reduce the numbers of chondrocytes in the superficial and intermediate zones of articular cartilage. To test this hypothesis, we adapted a stereological method [23] to determine chondrocyte cellularity in three dimensions [24, 25] and incorporated vital cell staining to compare the *chondrocyte volume fraction* in *Prg4* ^+/+^, *Prg4* ^+/-^, and *Prg4* ^-/-^ mice. By employing confocal microscopy to examine intact articular cartilage surfaces, we were able to avoid the need for histological processing and study larger volumes of articular cartilage. Herein, we describe our confocal imaging technique and report that *Prg4* ^-/-^ mice have significantly fewer articular cartilage chondrocytes than lubricin sufficient mice.

## Methods

All animal research was approved by the Rhode Island Hospital Animal Welfare Committee. The generation of mice with *Prg4* knockout alleles has been previously described [26]. In the present study, *Prg4* ^+/+^, *Prg4* ^+/-^, and *Prg4* ^-/-^ mice were maintained on the C57/Bl6 genetic background.

### Sample preparation

Hind limbs from *Prg4* ^+/+^, *Prg4* ^+/-^, and *Prg4* ^-/-^ mice of both sexes were harvested following CO_2_ asphyxiation at ten weeks of age (Additional file 1: Table S1). Left and right knee joints were dissected immediately after euthanasia in order to minimize chondrocyte death. The joint capsule was excised and soft tissue carefully removed to reveal the cartilaginous surface of the lateral and medial femoral condyles. Hind limbs were stained and analyzed by confocal microscopy or microCT (see below), or were fixed in 10 % phosphate buffered formalin (Fisher PROTOCOL™, Fisher Scientific, Waltham, MA, USA) and embedded in paraffin for activated caspase-3, terminal deoxynucleotidyl transferase dUTP nick end labeling (TUNEL) and 4′,6-diamidino-2-phenylindole (DAPI) histochemistry.

### Staining for confocal microscopy

**Fig. 1:**
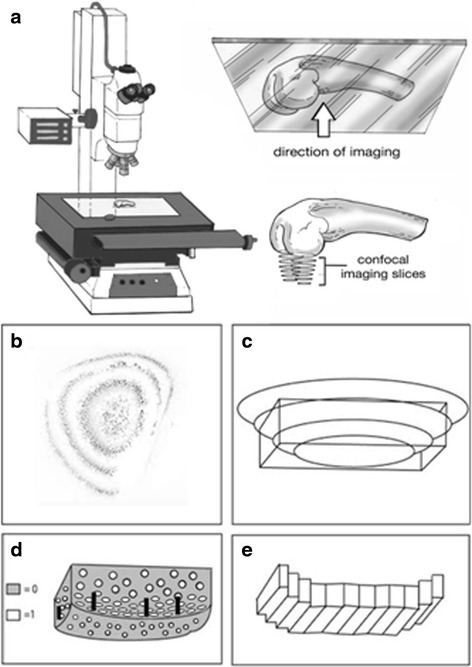
Schematic describing confocal imaging and processing technique. **a** Physical positioning of femur during imaging. **b** Subset of confocal image sections taken of the dome of a medial femoral condyle, looking from the apex downward in the z-direction (image inverted for clarity). **c** Region of interest (*rectangle*) selected from the 3D reconstruction of the condyle surface. **d** The method used to differentiate space occupied by cells and extracellular matrix. Areas of cellularity were labeled with “1” and non-cell areas were labeled with “0”. *Black bars* represent the vertical distance between areas of cellularity within the most superficial and deepest confocal images. Vertical distances were averaged across the width of the section to estimate the thickness of the superficial and upper intermediate zone. **e** Schematic demonstrating the rectangular prisms created iteratively and used to approximate the volume of the superficial and upper intermediate zone

Freshly dissected femoral condyles were stained using 1.3 μg/ml fluorescein diacetate (FDA) in PBS (Life Technologies, Cat # F1303), 0.1 μg/ml propidium iodide (PI) in PBS containing 2.5 % of EDTA (Sigma-Aldrich, Cat # P4170), and 1.16 μg/ml Hoescht 33342 trihydrochloride trihydrate in PBS (Life Technologies Cat # H33570) for 5 min in the dark at room temperature (Fig. 1). Samples then underwent five successive washes with PBS at room temperature and were placed facing downward on glass slides (Fig. 1a). Vectashield mounting medium (Vector Laboratories, Burlingame, CA, USA) was applied to the sample on the glass slide to prevent photo bleaching. FDA is a vital dye that only enters the cytoplasm of living cells. PI enters the cytoplasm of dead cells and binds to DNA. Hoechst stain enters live and dead cells, binds to DNA within the nucleus, and was used as a confirmatory stain for PI.

### Image acquisition using confocal microscopy

Confocal images from 0 to 28 μm in depth were acquired with a Nikon C1si confocal microscope (Nikon, Melville, NY, USA) using diode lasers 402, 488, and 561. Serial optical sections were performed with EZ-C1 computer software. Z-series sections were collected every 2 μm for a total of 14 images with a 10x PlanApo lens and a scan zoom of 1x. Each wavelength was acquired separately by invoking frame lambda. Imaging time for the stack was less than two minutes. Diode laser power was set to 10 % with a small pinhole. Projections were performed in Elements computer software (Nikon).

### Calculating cellularity and volume from the stacked confocal images

**Fig. 2:**
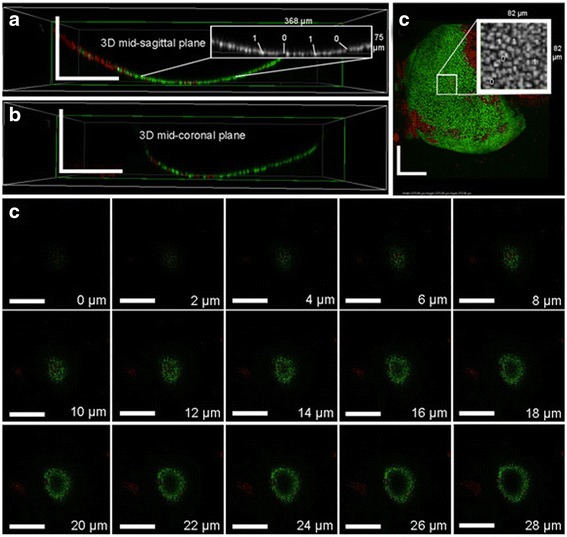
Representative 3D views showing reconstructed imaging of FDA and PI stained medial femoral condyle. **a** Mid-sagittal plane of reconstructed condylar images, including the pixel-labeling technique using 1s and 0s (*inset*). **b** Mid-coronal plane of reconstructed condylar images. **c** Reconstructed apical view of condyle with the pixel-labeling technique (*inset*). Scale bars indicate 250 μm. **d** Confocal z-stack image series showing sample sections from apex of condyle (0 μm) down into the medial femoral condyle cartilage thickness (28 μm). Estimated thickness of stained cartilage without loss of central cellularity was 14 μm, which included the superficial and upper intermediate cartilage zones. Scale bars indicate 400 μm. *FDA* fluorescein diacetate, *PI* propidium iodide

Images 0 – 14 μm in depth were processed using Matlab (Natick, MA, USA) (Fig. 1b) using a customized algorithm (Additional file 2: Figure S1). The depth was limited to 14 μm to restrict the analysis to the superficial and intermediate zones of weight bearing regions of mouse articular cartilage. A region of interest (ROI) approximating the weight-bearing surface of each condyle was selected from a three-dimensional reconstruction created from the image stacks (Fig. 1b, c). This ROI consisted of approximately the largest rectangular prism contained within the perimeter of the condyle (Fig. 1c). Monochrome images were thresholded to binary images with 1’s describing staining and 0’s describing absence of staining (Figs. 1d, 2a and c-insets). Images were then morphologically ‘skeletonized’ to ensure that each connected component had minimal weight and connectivity. This method was performed in order to normalize for differences in brightness, contrast, and staining luminescence between images, and ensure that each stained cell was described by an approximately equal number of pixels. Within this ROI, each vertical column of pixels in the z-direction was sampled to find the vertical distance between the lowest and highest cells, which was averaged in order to provide an approximate measure of thickness across the cartilage surface (Fig. 1e). Thickness was multiplied by the width of the ROI for each horizontal row, thus calculating the volume of the stained cartilage. Data for this calculation is limited by stain penetration and geometry of the curved surface. Images acquired had cells in the first few images and at 14 μm, a central loss of cellularity was observed when traversing downward through the z-stack, as shown in Fig. 2d. Thus, in order to obtain a reliable measure of thickness only the first eight images of the stack (14 μm of depth) were used. By counting how many sections were traversed, the vertical thickness of the stained layer could be determined, as sections were taken every 2 μm. The thickness so obtained was used for calculating the superficial + upper intermediate zone volume. This method draws upon one of the stereological tools in microscopy, the Cavalieri’s principle [27] which establishes that an unbiased measure of volume can be obtained by multiplying the thickness with area. The area here is that occupied and bound by chondrocytes in each slice.

The chondrocyte volume fraction for each condyle was calculated by counting the number of pixels labeled 1 (corresponding to cell staining) on each slice within the previously described ROI. The Holmes effect (i.e., overestimation of area occupied by cells on a slice) as described in the review by Mandarim-de-Lacerda [24], was avoided by using thin optical sections as recommended by Aherne and Dunhill [28], eliminating the need for compensating for average diameter measurements when using tissue sections. This “cell” volume was divided by the volume of the superficial and upper intermediate zone, based on the 14 μm depth, in order to find the percent of volume attributable to chondrocyte cellularity for each condyle. The chondrocyte volume fraction is equivalent to the stereological volume fraction described by Howard and Reed [29]. The “reference trap” was thus avoided by establishing reference volume a priori which was calculated the same way across all specimens [23].

### Cartilage thickness measurement using microCT imaging to assess the reference volume change

Mouse femur samples were prepared as described above and incubated in Hexabrix (Guerbet, Bloomington, IN, USA) contrast agent (15 % (v/v) in PBS) for 30 min at room temperature before scanning, which allowed improved contrast between bone and cartilage [30]. The femur was fixated using agarose in an inverted conical tube that was inserted into the 12.3 mm diameter microCT holder. Samples were imaged in air using 6 μm isotropic voxels at 45 kVp, 177 mA and 300 ms integration time (Scanco μCT 40, Bassersdorf, Switzerland). The epiphyseal region of the femur was imaged resulting in a scan time of approximately 45 min. Cartilage was segmented from the microCT scans using Mimics software (Mimics®, Materialise, Leuven, Belgium). The epiphyseal region of the bone was divided into the medial and lateral compartments using the patellofemoral groove as the midline. A predefined cube was created in Mimics software and was used to outline the most loadbearing region of cartilage for all the samples. A custom Matlab code was used to determine the thickness.

### Activated caspase-3 immunohistochemistry

Coronal mouse knee sections were heated to 60 °C for 30 min, deparaffinized and hydrated in three changes of xylene and serial alcohol. Antigen retrieval was performed using a pepsin solution (Thermo Fisher Scientific, Tewksbury, MA, USA) for 30 min. A rabbit polyclonal antibody against active caspase-3 (cat#ab13847, Abcam, Cambridge, MA, USA) at 1:100 dilution with 8 % horse serum in PBS was added to the slides and incubated at 4 °C overnight. After incubating with a Cy3 goat anti-rabbit IgG (Life Technologies, Catalog# A10520) at 1:100 dilution for one hour at room temperature in the dark, the sections were washed five times using PBS and coverslipped with vectashield mounting medium with DAPI (Vector Laboratories Inc., Burlingame, CA, USA). Images were captured at 20x with Image-Pro Plus software (Media Cyberkinetics, Bethesda, MD, USA).

### TUNEL staining

A TUNEL assay was performed on coronal mouse knee sections using an ApopTag Plus Peroxidase in situ Apoptosis Kit (Millipore, Billerica, MA, USA). Sections were heated at 60 °C for 30 min, deparaffinized in three changes of xylene and serial ethanol, then pretreated with proteinase K (20 μg/ml) for 15 min at room temperature, quenched with endogenous peroxidase in 3 % hydrogen peroxide for 5 min, and incubated with kit equilibration buffer for 30 s. Excess liquid was tapped off, and the sections were treated with terminal deoxynucleotidyl transferase enzyme at 37 °C for one hour in a humidified chamber. Sections were then washed three times in PBS, incubated with anti-digoxigenin conjugate for 30 min at room temperature, and washed in PBS. Peroxidase substrate was applied to sections, which were stained for 8 min., washed in deionized water, and counterstained with 0.5 % methyl green. Sections were washed in deionized water again, dehydrated in xylene three times, and mounted with Permount mounting media. Images were captured at 20x with Image-Pro Plus software.

### Two-dimensional cell counting

Images of thin sections stained with DAPI or probed for activated caspase-3 were acquired at the same exposure levels in all channels. They were then imported into Adobe Photoshop CS5 software and shadow levels in each channel were adjusted to eliminate background by modifying the histogram using the same values for all the images. Cell counting was performed using ImageJ software. A grid was overlaid on the image, starting at the condyle edge with horizontal lines 14 μm apart. Images for TUNEL were acquired in bright field with the same exposure and white balance settings and cell counting was performed as described above.

### Statistical analysis

Percent cellularity, cartilage thickness, and cell number are presented as mean ± SD*.* Differences in percent cellularity in a volume, cartilage thickness, and chondrocyte number data across genotypes were analyzed using a two-way repeated measures ANOVA (α ≤0.05). Tukey’s multiple comparison post hoc tests were used on all data. All statistical analyses were performed using Prism 6 (GraphPad Software Inc., San Diego, CA). The median value images of cellularity and cartilage thickness are depicted.

## Results

### Chondrocyte volume fractions determined by FDA stained volume/reference volume for the medial and lateral femoral condyles from wild-type and Prg4 mutant mice

**Fig. 3:**
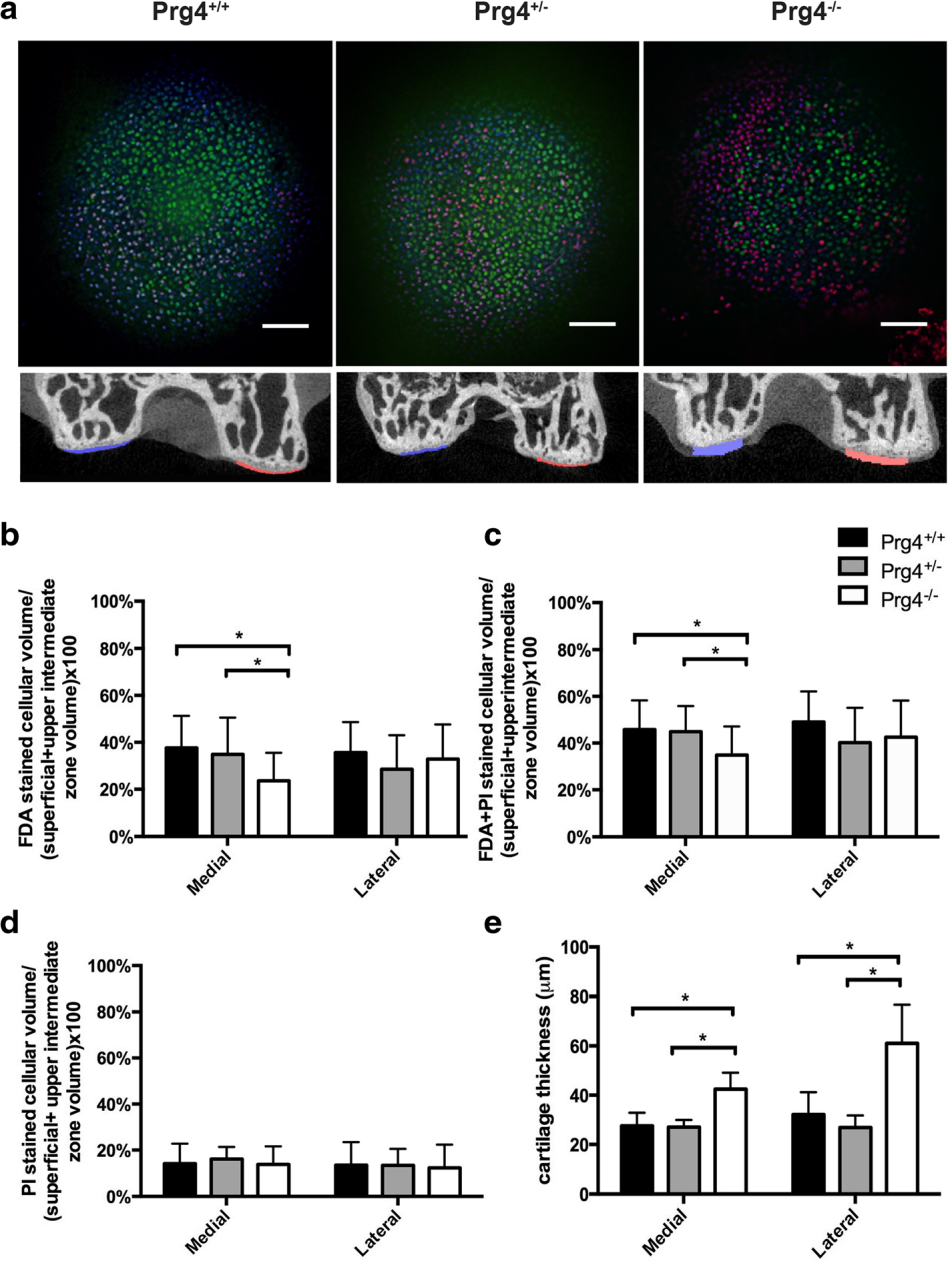
*Chondrocyte volume fraction* and *cartilage thickness* measurements medial and lateral femoral condyle articular cartilage across *Prg4* ^+/+^, *Prg4* ^+/-^ and *Prg4* ^-/-^ mouse knee joints. **a** Representative median confocal images of FDA (*green*) + PI (*red*) + Hoechst (*blue*) stained medial femoral condyle cartilage presented as a 0 to 14 μm depth projection, (*first row)* used in the calculation of mean percentage of cellular volume in cartilage*.* Scale bars indicate 10 μm. Representative microCT coronal sections (*second row*) illustrating the results of the Mimics cartilage reconstruction. **b** Superficial and upper intermediate zone living chondrocyte volume fraction (FDA stained cellular volume/superficial + upper intermediate zone volume) measurements for the medial and lateral femoral condyles. Living chondrocyte volume fraction of the medial femoral condyle of *Prg4* ^-/-^ mice was significantly less than *Prg4* ^+/+^ (*p* = 0.002) and *Prg4* ^+/-^ (*p* = 0.002) littermates. **c** Superficial and upper intermediate zone living + dead chondrocyte volume fraction (FDA + PI stained cellular volume/superficial + upper intermediate zone volume) measurements for the medial femoral lateral femoral condyles. Chondrocyte volume fraction of the medial femoral condyle of *Prg4* ^-/-^ mice was significantly less than *Prg4* ^+/+^ (*p* = 0.02) and *Prg4* ^+/-^ (*p* = 0.04) littermates. **d** Superficial and upper intermediate zone dead chondrocyte volume fraction (PI stained cellular volume/superficial + upper intermediate zone volume) measurements for medial and lateral femoral condyles. No significant differences were observed between genotypes on medial and lateral condyles. **e** Mean cartilage thickness determined from microCT, for the medial (*blue*) and lateral (*red*) femoral condyle are shown. Cartilage was significantly thicker in *Prg4* ^-/-^ medial femoral cartilage compared to *Prg4* ^+/+^ (*p* = 0.03) and *Prg4* ^+/-^ (*p* = 0.02) littermates. There was a significant increase in thickness for the lateral femoral condyle in *Prg4* ^-/-^ compared to *Prg4* ^+/+^ (*p* < 0.0001) and *Prg4* ^+/-^ (*p* < 0.0001) littermates. FDA fluorescein diacetate, *PI* propidium iodide

A representative femoral condyle stained with FDA, PI and Hoechst is shown in Fig. 3a. Qualitatively, there appeared to be more PI staining in *Prg4* ^*-/-*^ mice. Staining with FDA across all three genotypes showed stain penetration by depth (Fig. 2) as well as across the face of the condylar cartilage (Fig. 3). The superficial and upper intermediate zone living chondrocyte volume fractions defined as the ratio of FDA-stained (live/green) cellular volume/total superficial and upper intermediate zone (reference) volume for the medial femoral condyle for lubricin wild type (*Prg4* ^+/+^), heterozygote (*Prg4* ^+/-^), and knockout mice (*Prg4* ^-/-^) were 37.6 ± 13.7 % (*N* = 20 knee joints), 31.1 ± 14.3 % (*N* = 18), and 23.6 ± 11.9 % (*N* = 30), respectively. The *Prg4* ^-/-^ mice showed reduced cellularity compared to *Prg4* ^+/+^ (*p* = 0.002) and *Prg4* ^+/-^ (*p* = 0.002) littermates (Fig. 3b). There was no significant difference in cellularity between *Prg4* ^+/+^ and *Prg4* ^+/-^ mice (*p* = 0.82). In the lateral femoral condyle, living chondrocyte volume fractions for *Prg4* ^+/+^, *Prg4* ^+/-^ and *Prg4* ^-/-^ mice were 35.6 ± 13.1 % (*N* = 20 knee joints), 26.9 ± 12.9 % (*N* = 18), and 30.2 ± 12.4 % (*N* = 30), respectively. No significant differences were observed across genotypes, *Prg4* ^+/+^ vs. *Prg4* ^+/-^ (*p* = 0.27), *Prg4* ^+/-^ vs. *Prg4* ^-/-^ (*p* = 0.55), *Prg4* ^+/+^ vs. *Prg4* ^-/-^ (*p* = 0.78) (Fig. 3b).

### Chondrocyte volume fraction determined by FDA + PI stained volume/reference volume for the medial and lateral femoral condyles from wild-type and Prg4 mutant mice

The superficial and upper intermediate zone chondrocyte volume fractions defined as the ratio of FDA + PI-stained (living + dead) cellular volume/total superficial and upper intermediate zone (reference) volume for the medial femoral condyle of lubricin wild type (*Prg4* ^+/+^), heterozygote (*Prg4* ^+/-^), and knockout mice (*Prg4* ^-/-^) were 45.8 ± 12.6 % (*N* = 20 knee joints), 44.9 ± 11.1 % (*N* = 18), and 34.9 ± 12.2 % (*N* = 30), respectively. The *Prg4* ^-/-^ mice showed reduced cellularity compared to *Prg4* ^+/+^ (*p* = 0.02) and *Prg4* ^+/-^ (p = 0.04) littermates (Fig. 3c). There was no significant difference in cellularity between *Prg4* ^+/+^ and *Prg4* ^+/-^ mice (p = 0.98). For the lateral femoral condyle, total chondrocyte volume fractions for *Prg4* ^+/+^, *Prg4* ^+/-^ and *Prg4* ^-/-^ mice were 49.1 ± 13.1 % (*N* = 20 knee joints), 40.2 ± 14.9 % (*N* = 18), and 42.5 ± 15.7 % (*N* = 30), respectively, and were not significantly different, *Prg4* ^+/+^ vs. *Prg4* ^+/-^ (*p* = 0.11), *Prg4* ^+/-^ vs. *Prg4* ^-/-^ (*p* = 0.84), *Prg4* ^+/+^ vs. *Prg4* ^-/-^ (*p* = 0.22) (Fig. 3c).

### Chondrocyte volume fraction limited to the PI stained volume/reference volume for the medial and lateral femoral condyles from wild-type and Prg4 mutant mice

Mean superficial and upper intermediate zone dead chondrocyte volume fractions measurements defined as the ratio of PI-stained (dead/red) cellular volume/total superficial and upper intermediate zone volume for the medial femoral condyle for lubricin wild type (*Prg4* ^+/+^), heterozygote (*Prg4* ^+/-^), and knockout mice (*Prg4* ^-/-^) were 14.1 ± 8.7 % (*N* = 20 knee joints), 16.1 ± 5.3 % (*N* = 18), and 13.8 ± 7.8 % (*N* = 30), respectively (Fig. 3d). No significant differences in dead chondrocyte cellularity were observed between groups, *Prg4* ^+/+^ vs. *Prg4* ^+/-^ (*p* = 0.75), *Prg4* ^+/-^ vs. *Prg4* ^-/-^ (*p* = 0.62), *Prg4* ^+/+^ vs. *Prg4* ^-/-^ (*p* = 1.00). For the lateral femoral condyle, dead chondrocyte cellularity for *Prg4* ^+/+^, *Prg4* ^+/-^ and *Prg4* ^-/-^ mice was 13.5 ± 10.0 %, 13.4 ± 7.1 %, and 12.3 ± 10.0 %, respectively. No significant differences were observed across genotypes, *Prg4* ^+/+^ vs. *Prg4* ^+/-^ (*p* = 1.00), *Prg4* ^+/-^ vs. *Prg4* ^-/-^ (*p* = 0.91), *Prg4* ^+/+^ vs. *Prg4* ^-/-^ (*p* = 0.89) (Fig. 3d).

### Percent of FDA stained chondrocyte volume/FDA + PI stained volume for the medial and lateral femoral condyles from wild-type and Prg4 mutant mice

Mean percent of FDA-positive (live/green) chondrocyte volumes by confocal microscopy divided by FDA + PI stained volume in the superficial and upper intermediate zone (not corrected for superficial and upper intermediate zone volume) for the medial femoral condyle for *Prg4* ^+/+^, *Prg4* ^+/-^, and *Prg4* ^-/-^ mice were 61.9 ± 26.9 % (*N* = 20 knee joints), 54.6 ± 18.7 % (*N* = 18), and 63.0 ± 18.9 % (*N* =30), respectively. There were no significant differences between *Prg4* ^+/+^ and *Prg4* ^+/-^ (*p* = 0.07), and *Prg4* ^+/+^ and *Prg4* ^-/-^ (*p* = 0.98) littermates, but differences were observed between *Prg4* ^+/-^ and *Prg4* ^-/-^ individuals (*p* = 0.03). In the lateral femoral condyle mean percent of FDA-positive (live/green) staining of chondrocytes by confocal microscopy divided by FDA + PI stained volume in the superficial and upper intermediate zone (not corrected for superficial and upper intermediate zone volume) for *Prg4* ^+/+^, *Prg4* ^+/-^, and *Prg4* ^-/-^ mice was 72.6 ± 19.0 % (*N* = 20 knee joints), 63.9 ± 17.7 % (*N* = 18), and 70.9 ± 18.7 % (*N* = 30), respectively. There were no significant differences between genotypes in the lateral condyle, *Prg4* ^+/+^ vs. *Prg4* ^+/-^ (*p* = 0.39), *Prg4* ^+/-^ vs. *Prg4* ^-/-^ (*p* = 0.48), and *Prg4* ^+/+^ vs. *Prg4* ^-/-^ (*p* = 0.96).

### Cartilage thickness measurements across wild-type and Prg4 mutant mice

Mean cartilage thicknesses determined from the MIMICS reconstructions for the microCT scans of *Prg4* ^+/+^, *Prg4* ^+/-^ and *Prg4* ^-/-^ mice (*N* = 5 joints per genotype) for the medial femoral condyle were 27.6 ± 1.6 μm, 27.0 ± 1.4 μm, and 42.4 ± 2.6 μm, and for the lateral femoral condyle were 32.1 ± 1.9 μm, 26.8 ± 2.1 μm, and 61.0 ± 4.3 μm, respectively. A significant increase in *Prg4* ^-/-^ medial femoral condyle cartilage thickness was observed compared to *Prg4* ^+/-^ (*p* = 0.02) and *Prg4* ^+/+^ (*p* = 0.03) mice. No significant differences in medial condyle cartilage thickness were observed between *Prg4* ^+/+^ and *Prg4* ^+/-^ littermates (*p* = 1.00). A significant increase in *Prg4* ^*-/-*^ lateral condyle cartilage thickness was also observed compared to *Prg4* ^+/+^ (*p* < 0.0001) and *Prg4* ^+/-^(*p* < 0.0001) littermates. No significant differences in lateral condyle cartilage thickness were observed between *Prg4* ^+/+^ and *Prg4* ^+/-^ littermates (*p* = 0.60) (Fig. 3e).

### Caspase-3 activation in Prg4 null mice

**Fig. 4:**
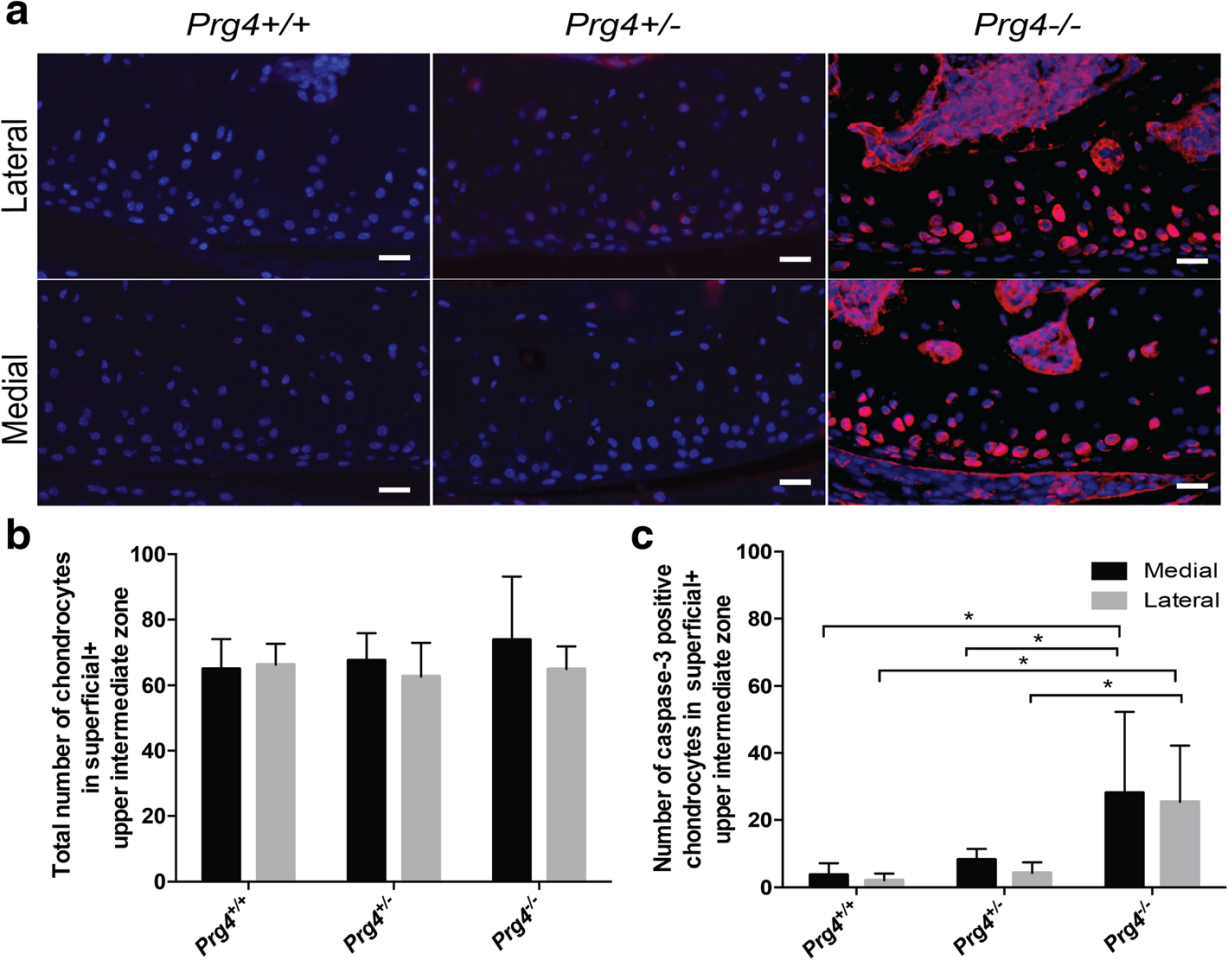
**a** Coronal sections of ten-week-old *Prg4* mutant mouse medial and lateral femoral condyles stained positive for activated capsase-3. Cells positive for activated caspase-3 are stained *pink*, while negative nuclei are *blue*. **b** No significant differences were observed in the total number of chondrocytes in superficial and upper intermediate zones (14 μm depth from the edge of the condyle) for *Prg4* ^+/+^, *Prg4* ^+/-^ and *Prg4* ^-/-^ littermates. **c** *Prg4* ^-/-^ femoral condyle cartilage had a significantly higher number of cells positive for activated caspase-3 in the superficial and upper intermediate zones (14 μm depth from the edge of the condyle) in the medial femoral condyle compared to *Prg4* ^+/-^ (*p* = 0.04) and *Prg4* ^+/+^(*p* = 0.01) cartilage. There was a significantly higher number of caspase positive cells in lateral femoral condyle in *Prg4* ^-/-^ mice compared to *Prg4* ^+/-^ (*p* = 0.02) and *Prg4* ^+/+^(*p* = 0.01) littermates. Scale bars indicate 20 μm

The mean total number of caspase-3 positive chondrocytes in superficial and upper intermediate zone (condyle edge to 14 μm depth) for *Prg4* ^+/+^ (*N* = 5 knee joints), *Prg4* ^+/-^ (*N* = 4), and *Prg4* ^-/-^(*N* = 5) medial femoral condyle were 3.7 ± 3.0, 8.1 ± 2.8 and 28.1 ± 21.6, respectively. Similarly, for the lateral femoral condyle, the total number of caspase-3 positive chondrocytes in superficial and upper intermediate zone for *Prg4* ^+/+^, *Prg4* ^+/-^ and *Prg4* ^-/-^ were 2.1 ± 1.8, 4.2 ± 2.7, and 25.4 ± 15.0, respectively. There was a significantly higher number of caspase-3 positive chondrocytes in *Prg4* ^-/-^ mouse medial femoral condyle cartilage compared to *Prg4* ^+/+^ (*p* = 0.01) and *Prg4* ^+/-^ (*p* = 0.04) littermates. Similarly, there was a significantly higher number of caspase-3 positive chondrocytes in *Prg4* ^-/-^ mouse lateral femoral condyle compared to *Prg4* ^+/+^ (*p* = 0.01) and *Prg4* ^+/-^ (*p* =0.02) littermates (Fig. 4a, c).

### TUNEL staining

The mean total number of TUNEL positive chondrocytes in superficial and upper intermediate zone (condyle edge to 14 μm depth) for *Prg4* ^+/+^ (*N* = 5 knee joints), *Prg4* ^+/-^ (*N* = 4) and *Prg4* ^-/-^ (*N* = 5) medial femoral condyle were 3.9 ± 4.1, 9.6 ± 6.4, and 5.7 ± 6.7, respectively and lateral femoral condyle were 5.9 ± 4.8, 2.0 ± 1.8, and 5.2 ± 7.2. There were no significant differences in the number of TUNEL positive cells in the superficial and upper intermediate zone among different genotypes (*p* > 0.05 for all comparisons).

### Traditional thin section cell counting in wild-type and Prg4 mutant mice

The total number of chondrocytes in the superficial and upper intermediate zones (condyle edge to 14 μm depth) for *Prg4* ^+/+^ (*N* = 5 knee joints), *Prg4* ^+/-^ (*N* = 4), and *Prg4* ^-/-^ (*N* = 5) medial femoral condyle were 64.9 ± 8.2, 67.5 ± 7.2, and 73.8 ± 17.3, respectively. For the lateral femoral condyle, the total number of chondrocytes in the superficial and upper intermediate zones (condyle edge to 14 μm depth) for *Prg4* ^+/+^, *Prg4* ^+/-^ and *Prg4* ^-/-^ were 66.3 ± 5.7, 62.7 ± 9.2, and 64.9 ± 6.2, respectively. No significant differences were observed between groups (*p* >0.05 for all comparisons) (Fig. 4b).

## Discussion

Though CACP is a rare disorder, it is valuable in understanding the etiology of a number of arthritic conditions, including post-traumatic OA (PTOA) [31–33]. The lubricin knockout mouse recapitulates most of the clinical features of CACP [6]. Evidently, this includes the loss of superficial zone chondrocytes, which is clinically relevant, since patients with CACP require joint replacement surgery by the third or fourth decade. Lubricin acts as a boundary lubricant, decreasing the friction coefficient in joints at near-zero sliding speeds in order to counteract the shear-induced tissue strain associated with normal articulation [4, 5]. The synovial fluid from patients with CACP has no lubricin and is unable to reduce friction in vitro in a mechanical bearing comprised of latex apposed to glass [4]. High friction induces chondrocyte caspase-3 activation in lubricin null mice. However, whether this translates into cellular loss was unknown [8, 34].

Staining of articular cartilage with FDA was previously used to confirm chondrocyte viability across *Prg4* ^+/+^, *Prg4* ^+/-^ and *Prg4* ^-/-^ murine knee joints that had been cyclically loaded ex vivo for up to 38 hrs [18]. In that work, and presently, we were unable to find any correlation between *Prg4* genotype and the percentage of live cells in the superficial and upper intermediate zone using FDA and standard fluorescence microscopy in *two* dimensions. However, by measuring the volume of living chondrocytes (green) and combining these data with the volume of dead chondrocytes (red) divided by the cartilage tissue volume for each genotype, our method yielded a reliable metric of superficial and upper intermediate zone cellularity as a *chondrocyte volume fraction*. A similar stereoscopic metric has been described in a thin section of bovine cartilage imaged by confocal microscopy but that method used a ‘dissector’ and was semi-automated [35] in that the cells were counted manually by placing a grid in each image of the z-stack. Cell volume *fraction* has been used in other tissues as well, especially in the brain [36]. The ROI in our study was limited to a depth of 14 μm in mouse cartilage from the cartilage surface origin. Figure 2d shows a loss of central cellularity in the z-stack at 14 μm depth. Thus, digitized tissue volumes imaged up to that depth would be representative of cell and extracellular volume. The fractional value of cell volume fraction we observed, ~ 40 % is greater than 1.65 - 3.0 % reported for human cartilage [37] and exceeds those of larger quadrupeds [38].

We chose to calculate cellular volume fraction in two ways: 1) using the volume of FDA stained cells in the numerator or 2) the volume of FDA + PI stained cells in the numerator, since there was no prior knowledge regarding the rate of chondrocyte demise in CACP in humans or in mice. As shown in Fig. 3b and c, similar results were observed for both approaches leading to the conclusion that there are fewer chondrocytes in the superficial and intermediate zone in *Prg4* ^-/-^ (lubricin null) cartilage. This result was strengthened upon measuring an increase in cartilage thickness by microCT. Cartilage from *Prg4* ^-/-^ mice appears thicker than that from Prg*4* ^+/-^ and *Prg4* ^+/+^ littermates, which is not due to a thickened lamina splendens formed by serum proteins which biofoul the articular surface in the absence of lubricin [4, 39]. The lateral and medial femoral condyle cartilage of *Prg4* ^-/-^ mice was respectively 47.4 % and 35.1 % thicker compared to *Prg4* ^+/+^ littermates. *Prg4* ^+/-^ mice had a non-significant decrease in thickness, 2 % for medial and 20 % for lateral femoral condyle, compared to *Prg4* ^+/+^ littermates. However, the reduction in chondrocyte volume fraction was confined to the medial femoral condyle, the major load bearing joint compartment. The multidimensional increase in volume via cartilage swelling does not explain the reduction in chondrocyte volume fraction, since the lateral femoral condyle showed no loss of cell volume fraction. Animal weight is unaffected by these *Prg4* mutations [40] and thus would not account for these differences. Cartilage swelling in the medial compartment following injury has been observed previously in a cohort of patients with ACL injury and reconstruction [41] and is not unique to lubricin insufficient mice. The majority of joint loading occurs in the medial articular surfaces of most mammalian joints [42, 43]. Lubricin deficiency, whether genetic or transient, will result in mechanical strain [7] and apoptosis [8] of subsurface chondrocytes which we observed. Chondrocytes displaying activated caspase-3 may not ultimately become TUNEL positive. Recently dead cells will stain with PI. However, the chondrocyte volume fraction calculation, limited to the PI stained volume, divided by the reference volume, for both femoral condyles failed to detect more dead chondrocytes in cartilage from *Prg4* ^*-/-*^ mice compared to *Prg4* sufficient littermates. Based upon our results we posit that a portion of unstained extracellular volume was *once occupied* by chondrocytes in younger *Prg4* ^-/-^mice and would thus also be non-detectable by TUNEL staining. Many chondrocytes in adult *Prg4* ^-/-^ mice display caspase-3 activation and yet these mice do not show a rapid and precipitous joint failure, leading us to also speculate that other mechanisms [44] may exist to inhibit caspase-3. These possibilities may explain our observation that there were no differences in TUNEL positivity across the three *Prg4* genotypes.

FDA staining has been established as a robust method for determination of cell viability with consistent viability values measured over time [45]. FDA being a nonpolar compound easily enters the lipid bilayer of viable cell membranes and is hydrolyzed by cytosolic esterases producing fluorescein [46]. Fluorescein, being a polar compound, is not extruded rapidly as this ester becomes accumulated in the cytoplasm, which allows for robust imaging and cell volume determination [46]. Residual esterase activity has been observed in dying pancreatic islet cells producing faint green fluorescence [47]. Frame lambda mode imaging and image processing have compensated for any imperceptible green staining of dying chondrocytes, if present. The combined depth of the superficial, intermediate and deep zones in mouse femoral condyle cartilage is 25 μm [48]. The present study is limited by our inability to report actual chondrocyte cell number, but provides a useful and accurate approximation by instead calculating chondrocyte volume fraction in a larger volume occupied by both cells and extracellular matrix - a measure of cell volume density. By contrast, the total number of chondrocytes in the superficial and upper intermediate zone calculated using a traditional method from the condyle edge to a depth of 14 μm (Fig. 4b) across the three genotypes showed no differences. Live/(live + dead) cell volume ratio calculation similarly did not indicate cellular loss in the medial femoral condyle of *Prg4* null mice.

In animal models of PTOA, damage to the articular cartilage matrix occurs concomitantly with a loss of boundary-lubricating ability of synovial fluid [9, 12]. The elevated coefficient of friction accompanying this loss occurs concurrently with decreased lubricin synthesis by synovial fibroblasts and superficial zone chondrocytes [12, 49]. Chondrocyte apoptosis is observed in both humans and animal models following mechanical injury and appears to be closely associated with the progression of cartilage degradation in OA [14, 16, 50–52]. Supplementing synovial fluid with lubricin [53] (tribosupplementation) limits cartilage degeneration in traumatic joint injury as evidenced by less collagen type II degradation in a rodent PTOA model, accompanied by an improvement in joint function in vivo [54–56]. Fewer chondrocytes undergo apoptosis and cartilage degeneration is reduced following tribosupplementation in rodent models of PTOA [56–58]. Decreased lubricin concentrations may correlate with increased production of inflammatory cytokines in guinea pig models of PTOA [59]. The results from this study, and others [8, 54, 57], indicate that in the absence of lubricin, there is an increase in the demise of superficial and upper intermediate zone chondrocytes.

Limitations of this study include the restriction of mouse age to ten weeks. At that age mouse joints are fully developed and chondrocytes no longer divide which may have restricted our ability to detect greater numbers of cells that stained with PI. Hoechst was used as a confirmatory stain of PI staining of DNA. Fractional cell volume calculations were not performed using Hoechst staining alone since it appeared (Fig. 3a) that FDA competed with Hoechst stain. Future use of this stereoscopic method could be accomplished using Hoechst as the singular vital stain in the event that the proportion of live to dead cells is less relevant than the total cell volume fraction. However, both Hoechst and PI are limited as they will not stain the cytoplasm and thus may not provide a true estimate of cellular volume.

## Conclusions

In the absence of *Prg4*, there is an increase in the demise of superficial and upper intermediate zone chondrocytes, possibly through increased apoptosis in these zones. Cellular loss can be measured as a loss of stained cell volume compared to the total volume of the cartilage tissue imaged by confocal microscopy. We developed a novel approach to quantify superficial and upper intermediate zone cellularity in the intact bearing surface of mouse femoral condyles that does not require fixation. Imaging cartilage with a larger radius of curvature from other species would result in imaging of chondrocytes deeper in the z-stack before the central loss of cellularity as observed in Fig. 2d. The cartilage structure is not damaged during imaging, and could theoretically be mechanically tested either before or after imaging.

## Additional files

Additional file 1: Table S1. *Prg4* wild-type and mutant mice utilization for chondrocyte volume fraction determination of the medial and lateral femoral condyle by genotype, sex and knee laterality. (PDF 110 kb)

Additional file 2: Figure S1. Matlab script for chondrocyte volume fraction calculation. (PDF 110 kb)

## Abbreviations

CACP: Camptodactyly-arthropathy-coxa vara-pericarditis
DAPI: 4′,6-diamidino-2-phenylindole
FDA: Fluorescein diacetate
OA: Osteoarthritis
PI: Propidium iodide
PRG4: Lubricin
PTOA: Post-traumatic osteoarthritis
TUNEL: Terminal deoxynucleotidyl transferase nick end labeling
