# Supplementary material for: Superficial zone cellularity is deficient in mice lacking lubricin: a stereoscopic analysis
Source: Arthritis Res Ther. 2016 Mar 14;18:64. doi: 10.1186/s13075-016-0967-4 (PMC5477516; doi:10.1186/s13075-016-0967-4)
Supplement: Supplementary file 1 — Prg4 wild-type and mutant mice utilization for chondrocyte volume fraction determination of the medial and lateral femoral condyle by genotype, sex and knee laterality. (PDF 110 kb) [file 13075_2016_967_MOESM1_ESM.pdf]

Additional file1: Table S1. *Prg4* mutant mice utilization for chondrocyte volume fraction determination of the medial and lateral femoral condyle by genotype, sex and knee laterality.

Prg4 +/+ (N=20 knees)				*Prg4* +/- (N=18 knees)				*Prg4* -/- (N=30 knees)			
#	Sex	L	R	#	Sex	L	R	#	Sex	L	R
2863	♂		●	2880	♂		●	2851	♂	●	●
2864	♂	●		2881	♂	●		2852	♂		●
2865	♂		●	2886	♀	●		2872	♂	●	
2888	♀	●		3012	♂	●	●	3020	♂	●	●
3034	♀	●	●	3013	♂	●	●	3021	♂	●	●
3010	♀	●	●	3014	♂		●	3022	♂	●	●
3011	♀		●	3016	♂	●	●	3023	♂	●	●
3015	♂	●	●	3028	♂	●	●	3009	♀	●	●
2870	♀	●	●	3029	♂	●	●	3024	♀	●	●
2869	♀		●	57	♀	●	●	3026	♂	●	●
1	♂	●	●	63	♀	●	●	17	♀	●	●
2	♂	●	●					19	♀	●	●
3	♂	●	●					3095	♀	●	●
								65	♀	●	●
								3102	♀	●	●
								3103	♀	●	●
