# Supplementary material for: Superficial zone cellularity is deficient in mice lacking lubricin: a stereoscopic analysis
Source: Arthritis Res Ther. 2016 Mar 14;18:64. doi: 10.1186/s13075-016-0967-4 (PMC5477516; doi:10.1186/s13075-016-0967-4)
Supplement: Supplementary file 2 — Matlab script for chondrocyte volume fraction calculation. (PDF 110 kb) [file 13075_2016_967_MOESM2_ESM.pdf]

```
%Cellularity%
% enter the inputs- first and the last image number from the stack,filename and threshold%
%thresholding%
imgstart=56;
imgend=63;
totalim=imgend-imgstart;
green=zeros(512,512,totalim);
red=zeros(512,512,totalim);
for i=1:(totalim+1)
    percent=i/(totalim+1)*100;
    progress=strcat('processing...', num2str(percent,2),'%');
    figure(1),title(progress);
    h=gcf;
    set(h,'outerposition',[100,800,800,800]);

    green_temp_string=strcat('2880 l-1z',num2str(imgstart+i-1,'%02.0f'),'c2.tif');
    red_temp_string=strcat('2880 l-1z',num2str(imgstart+i-1,'%02.0f'),'c3.tif');
    green_temp=imread(green_temp_string);
     red_temp=imread(red_temp_string);
    figure(1)
    subplot(2,2,1);
    imagesc(green_temp); colormap gray; hold on;
    axis image; title(strcat('green',num2str(i)));
    subplot(2,2,2);
    imagesc(red_temp); colormap gray; hold on;
    axis image; title(strcat('red',num2str(i)));
    imthresholdg=0.15;
    imthresholdr=0.2;
    lvlgreen=min(min(green_temp))+imthresholdg*(-min(min(green_temp))+max(max(green_temp)));
    lvlred=min(min(red_temp))+imthresholdr*(-min(min(red_temp))+max(max(red_temp)));
    green_temp=(green_temp>lvlgreen);
    red_temp=(red_temp>lvlred);
    green(:,:,i)=green_temp;
    red(:,:,i)=red_temp;
end; g='green';
r='red';
save(g,'green');
save(r,'red');
%display the stack projected as a 2D image%
sum_image=zeros([512 512 1]);
gsize=size(green);
for m=1:5:gsize(3)
    sum_image=sum_image|green(:,:,m);
    sum_image=sum_image|red(:,:,m);
end
figure(1);clf;
imagesc(sum_image);colormap gray;hold on;
title('sum image');
text(10,20,'select ROI: upper left then lower right','color','white');
[cropx1,cropy1]=ginput(1);
cropx1=floor(cropx1);
cropy1=floor(cropy1);
```

```
plot([1,512],[cropx1 cropy1],'-r');
plot([cropx1 cropy1],[1 512],'-r');
[cropx2, cropy2]=ginput(1);
cropx2=floor(cropx2);
cropy2=floor(cropy2);
plot([1,512],[cropx2 cropy2],'-r');
plot([cropx2 cropy2],[1 512],'-r');

%finding thickness

slicel=cropx1;
slicer=cropx2;
slicet=cropy1;
sliceb=cropy2;
width=slicer-slicel;
gsize=size(green);
thick=gsize(3)-1;
% get top and bottom layer of surface, tag the pixels with staining as 1 in respective channels%
%calculate the thickness and volume%
green_crop=green(slicet:sliceb,slicel:slicer,(gsize(3)-thick):gsize(3));
red_crop=red(slicet:sliceb,slicel:slicer,(gsize(3)-thick):gsize(3));
both_crop=green_crop|red_crop;
siz=size(both_crop);
both_mins=zeros(siz);
both_mins_flat=zeros([512 width 1]);
both_maxs=zeros(siz);
both_maxs_flat=zeros([512 width 1]);
thickness=[];
row_aggregate=[];
v=0;
r=0;
for i=1:siz(1)
    row=[];
    for j=1:siz(2)
        col=find(both_crop(i,j,:)>0);
       if(isempty(col)==0)
           bmin=min(col);
           bmax=max(col);
           both_mins(i,j,bmin)=1;
           both_maxs(i,j,bmax)=1;
           both_mins_flat(i,j,:)=bmin;
           both_maxs_flat(i,j,:)=bmax;
           if(bmin~=bmax && bmin~=0&& bmax~=0)
               col_aggregate={col;col};
               thickness=[thickness;(bmax-bmin)];
               row=[row;(bmax-bmin)];
           end
       end
    end
        if(isempty(row)==0)
            row_aggregate=[row_aggregate;mean(row)];
            v=v+(mean(row)*(slicer-slicel));
            r=r+1;
        end
```

```
end
%green cellularity
green_num=size(find(green_crop>0));
%red cellularity
red_num=size(find(red_crop>0));
%total cellularity
total_num=green_num(1)+red_num(1);

green_num(1);
red_num(1);
total_num;
v; c_density=total_num/v*100;
green_percent=green_num(1)/total_num*100;
red_percent=red_num(1)/total_num*100;
mean(thickness);
```

```
%open a file named cellularity_data and write results in it%
cellularity_data={'green',green_num(1);'red',red_num(1);'total',total_num;'volume',v;
'cellularity',c_density;'green%',green_percent;'red%',red_percent;'thickness',mean(thickness)};
fileID=fopen('cellularity_data.dat','w');
formatSpec='%s %2.4f\n';
[nrows,ncols]=size(cellularity_data);
for row=1:nrows
    fprintf(fileID,formatSpec,cellularity_data{row,:});
end
fclose(fileID);
```

```
%morphologically skeletonize images, find centroid and circle the live cells in green%
gsize=size(green);
skel_green=zeros([512 512 gsize(3)]);
skel_red=zeros([512 512 gsize(3)]);
for i=1:gsize(3)
    %progress percent=i/(gsize(3)+1)*100;
    progress=strcat('processing...',num2str(percent,2),'%');
    figure(1),title(progress);
    h=gcf;
    set(h,'outerposition',[100,800,800,800]);

    skel_green(:,:,i)=bwmorph(green(:,:,i),'skel',Inf);
    skel_red(:,:,i)=bwmorph(red(:,:,i),'skel',Inf);

end
```

```
bcsize=size(both_crop);
```

```
%y slices(top to bottom)
for i=1:bcsize(2) figure(1);
    imshow(flipud(permute(both_crop(:,i,:),[3 1 2])))
    pause(.1);
end
```

```
bcsize=size(both_crop);

%x slices(side to side)
for i=1:bcsize(1) figure(1);
    imshow(flipud(permute(both_crop(i,:,:),[3 2 1])))
    pause(.1);
end green_cc=bwconncomp(green);
STAT=regionprops(green_cc,'centroid');
cents=struct2cell(STAT);
green_cells=[];
for i=1:length(cents)
    green_cells=[green_cells; cents{i}];
end
```

```
%reconstruct the condyle as 3D surface %
plot3(green_cells(:,1,:), green_cells(:,2,:),green_cells(:,3,:),'og');
daspect([1 1 1]);
axis vis3d;
```

Published with MATLAB® R2014a
